# Intraperitoneal alpha therapy with ^224^Ra-labeled microparticles combined with chemotherapy in an ovarian cancer mouse model

**DOI:** 10.3389/fmed.2022.995325

**Published:** 2022-10-10

**Authors:** Roxanne Wouters, Sara Westrøm, Yani Berckmans, Matteo Riva, Jolien Ceusters, Tina B. Bønsdorff, Ignace Vergote, An Coosemans

**Affiliations:** ^1^Laboratory of Tumor Immunology and Immunotherapy, Department of Oncology, Leuven Cancer Institute, KU Leuven, Leuven, Belgium; ^2^Oncoinvent AS, Oslo, Norway; ^3^Department of Neurosurgery, Mont-Godinne Hospital, UCL Namur, Yvoir, Belgium; ^4^Division of Gynecological Oncology, Department of Obstetrics and Gynecology, Leuven Cancer Institute, University Hospitals Leuven, Leuven, Belgium; ^5^Department of Oncology, Gynecological Oncology, KU Leuven, Leuven, Belgium

**Keywords:** ovarian cancer, alpha therapy, radium-224, chemotherapy, carboplatin, paclitaxel, pegylated liposomal doxorubicin

## Abstract

A novel alpha-therapy consisting of ^224^Ra-labeled calcium carbonate microparticles (^224^Ra-CaCO_3_-MP) has been designed to treat micrometastatic peritoneal disease via intraperitoneal (IP) administration. This preclinical study aimed to evaluate its efficacy and tolerability when given as a single treatment or in combination with standard of care chemotherapy regimens, in a syngeneic model of ovarian cancer in immune competent mice. Female C57BL/6 mice bearing ID8-fLuc ovarian cancer were treated with ^224^Ra-CaCO_3_-MP 1 day after IP tumor cell inoculation. The activity dosages of ^224^Ra ranged from 14 to 39 kBq/mouse. Additionally, ^224^Ra-CaCO_3_-MP treatment was followed by either carboplatin (80 mg/kg)-pegylated liposomal doxorubicin (PLD, 1.6 mg/kg) or carboplatin (60 mg/kg)-paclitaxel (10 mg/kg) on day 14 post tumor cell inoculation. All treatments were administered via IP injections. Readouts included survival, clinical signs, and body weight development over time. There was a slight therapeutic benefit after single treatment with ^224^Ra-CaCO_3_-MP compared to the vehicle control, with median survival ratios (MSRs) ranging between 1.1 and 1.3. The sequential administration of ^224^Ra-CaCO_3_-MP with either carboplatin-paclitaxel or carboplatin-PLD indicated a synergistic effect on overall survival at certain ^224^Ra activities. Moreover, the combinations tested appeared well tolerated in terms of weight assessment in the first 4 weeks after treatment. Overall, this research supports the further evaluation of ^224^Ra-CaCO_3_-MP in patients with ovarian cancer. However, the most optimal chemotherapy regimen to combine with ^224^Ra-CaCO_3_-MP should be identified to fully exploit its therapeutic potential.

## Introduction

Ovarian cancer is the eight leading cause of cancer related deaths within the female population worldwide (1, 2). The most dominant subtype is high-grade serous ovarian cancer (HGSOC), which has an epithelial origin (3, 4). Due to the absence of symptoms at earlier stages of the disease, patients are often diagnosed at an advanced disease stage (International Federation of Gynecology and Obstetrics (FIGO) stage III and IV). In first line, the current standard of care of advanced ovarian cancer consists of a cytoreductive debulking surgery combined with platinum-based chemotherapy and eventually bevacizumab and/or poly (ADP-ribose) polymerase (PARP) inhibitors (5). The carboplatin-paclitaxel chemotherapy combination is the preferred regimen in a first-line treatment setting. However, the choice of chemotherapy at recurrence depends on tumor characteristics and whether platinum is again an option or not. At the time of recurrence, both platinum and non-platinum agents such as paclitaxel, gemcitabine, pegylated liposomal doxorubicin (PLD) and topotecan, as well as targeted therapies such as PARP inhibitors and bevacizumab can be used as single agents or in combination schedules (6). Nevertheless, HGSOC patients who get diagnosed in an advanced disease stage have a poor 5 year survival of only 20%-41% (7).

In most patients, recurrence of the disease involves the presence of metastases confined to the peritoneal cavity. In the ongoing search for more effective treatment strategies to locally target this peritoneal disease, the rapidly evolving research field of radionuclide therapy is of interest. Historically, the main focus in the context of ovarian cancer was on the investigation of β-particle emitters, with various success rates. In the past, radiocolloids containing ^32^P or ^198^Au have been used for IP treatment of patients with ovarian cancer (8–11). However, its use was limited in time due to the increased incidence of adverse effects, and replacement with chemotherapy treatment was recommended (9). Additionally, antibody guided ^90^Y has been explored in the context of ovarian cancer, but it did not proceed from phase III clinical trials (12, 13). Alternative types of radiotherapy options that have been explored for ovarian cancer include proton beam therapy and carbon ion therapy with successful outcomes in two case reports of patients with recurrent ovarian cancer (14, 15).

The use of α-particle emitters is assumed to have advantages over the prior β-therapies. They are particularly of interest for the treatment of micrometastatic cancer dissemination in body cavities, one of the characteristics of peritoneal carcinomatosis in patients with ovarian cancer (16). Alpha-emitters are highly cytotoxic for the cancer cells residing in the abdominal cavity, while sparing surrounding radiosensitive organs because of their short penetration depth, thus limiting toxicities compared to β-emitters. To date, Xofigo^®^ (^223^RaCl_2_) remains the only α-emitting radiopharmaceutical approved by the European Medicines Agency and the Food and Drug Administration, and is currently used for the treatment of skeletal metastases of castration-resistant prostate cancer (17). However, α-emitters, such as ^212^Pb and ^211^At have been investigated for ovarian cancer in phase I clinical trials, where feasibility of this type of treatment was confirmed without apparent signs of dose-limiting toxicities (18, 19).

The α-emitter ^224^Ra, when adsorbed onto the microparticle drug carrier CaCO_3_, has shown therapeutic potential in immunodeficient ovarian cancer xenograft mouse models, when administered as an IP treatment (20, 21). Based on these promising data, the ^224^Ra-CaCO_3_-MP are currently being assessed in phase I clinical trials for both ovarian cancer (22) and colorectal carcinoma (23), two cancer types characterized by the presence of a widespread metastatic disease within the peritoneal cavity.

The current paper focusses on the evaluation of ^224^Ra-CaCO_3_-MP in terms of its potential to treat peritoneally disseminated ovarian cancer in an immune competent mouse model. The aim was to examine the tolerability and efficacy of combinations with chemotherapy regimens commonly used in patients with ovarian cancer.

## Materials and methods

### Ovarian cancer tumor model

The ID8-fLuc cell line was transduced with a lentiviral vector (pCHMWS_CMV-fluc-I-PuroR) by the Laboratory of Molecular Virology and Gene Therapy and Leuven Viral Vector Core in our institute (KU Leuven, Belgium) (24). Female C57BL/6 mice (Envigo, Horst, The Netherlands) of seven to 9 weeks of age were inoculated IP with 5 x 10^6^ ID8-fLuc ovarian cancer cells on day 0 of the experiment, in the lower right quadrant of the abdomen. All animal experiments were approved by the ethical committee of the KU Leuven (P123/2017) and followed the most recent ethical standards (NIH guidelines for the Care and Use of Laboratory Animals and EU Directive 2010/63/EU as amended by Regulation (EU) 2019/1010) and the ARRIVE (Animal Research: Reporting of In Vivo Experiments) guidelines (25, 26). All mice in experiment were monitored at least three times per week in terms of body weight and clinical signs of disease, and drained from ascites when mice reached 32 grams. When pre-defined humane endpoints were reached [previously published (24)], mice were euthanized by cervical dislocation.

### ^224^Ra-CaCO_3_-MP preparation and treatment in mice

Two product formulations of ^224^Ra-CaCO_3_-MP have been used: ^224^Ra-CaCO_3_-MP-1 from the early-phase of development and the optimized ^224^Ra-CaCO_3_-MP-2, developed for clinical use in humans. The preparation of the ^224^Ra-CaCO_3_-MP-1 product formulation is described as second generation microparticles (27), whereas the preparation of the ^224^Ra-CaCO_3_-MP-2 formulation can be found in a separate publication where they are described as layer-encapsulated microparticles (28). In brief, CaCO_3_ microparticles were prepared by a spontaneous precipitation method which yielded particles with a mainly spherical geometry with a volume-based median diameter of approximately 6 μm when measured by laser diffraction (Mastersizer 3000, Malvern Instruments Ltd, Worcestershire, UK). For radiolabeling, ^224^RaCl_2_ solution was added to a suspension of CaCO_3_ microparticles in the presence of Ba^2+^ and SO${}_{4}^{2-}$ (0.004% and 0.6% (*w*/*w*) relative to CaCO_3_ respectively) for the coprecipitation of ^224^Ra. After the radiolabeling process, the ^224^Ra-CaCO_3_-MP-1 were dispersed to a concentration of approximately 12.5 mg/ml in 0.9% NaCl. To fulfill the requirements for the clinical use of the radiopharmaceutical, it was necessary to control the size of microparticles in suspension over time and introduce a sterilization process. Hence, for the ^224^Ra-CaCO_3_-MP-2, an additional layer of CaCO_3_ was precipitated on the microparticles before they were dispersed to 25 mg/ml in 0.9% NaCl and 2.4% (*w/w*) EDTMPA [ethylenediamine tetra(methylenephosphonic acid)] and sterilized in an autoclave at 121 °C for 20 min. The ^224^Ra-CaCO_3_-MP-2 product was diluted to a final concentration of 12.5 mg/ml with Plasmalyte (Baxter, Deerfield, IL, USA) prior to treatment administration in mice. Radium-224 labeled microparticles were administered *via* an IP injection at a volume of 0.4 ml on day 1 post tumor cell inoculation at a mass dose of 5 mg CaCO_3_ and an activity dose ranging between 14 and 39 kBq/mouse ^224^Ra (805 and 2118 kBq/kg, respectively).

### Chemotherapy preparation and treatment in mice

Carboplatin and paclitaxel (Hospira, ONCO-TAIN, Pfizer, New York, NY, USA) were dissolved in Dulbecco's phosphate-buffered saline (DPBS, Thermo Fisher Scientific, Waltham, MA, USA) and administered IP at a dose of 60 or 80 mg/kg and 10 mg/kg, respectively, calculated for an average body weight of 20 g per mouse. Pegylated liposomal doxorubicin (Caelyx/Doxil^®^, Janssens Cilag International NV, Beerse, Belgium) was administered IP at a dose of 1.6 mg/kg. All chemotherapy doses used in this manuscript were determined previously via *in vivo* dosage experiments for each of the combination schedules in the ID8-fLuc mouse model for ovarian cancer (unpublished results).

### Experimental design

**Figure 1:**
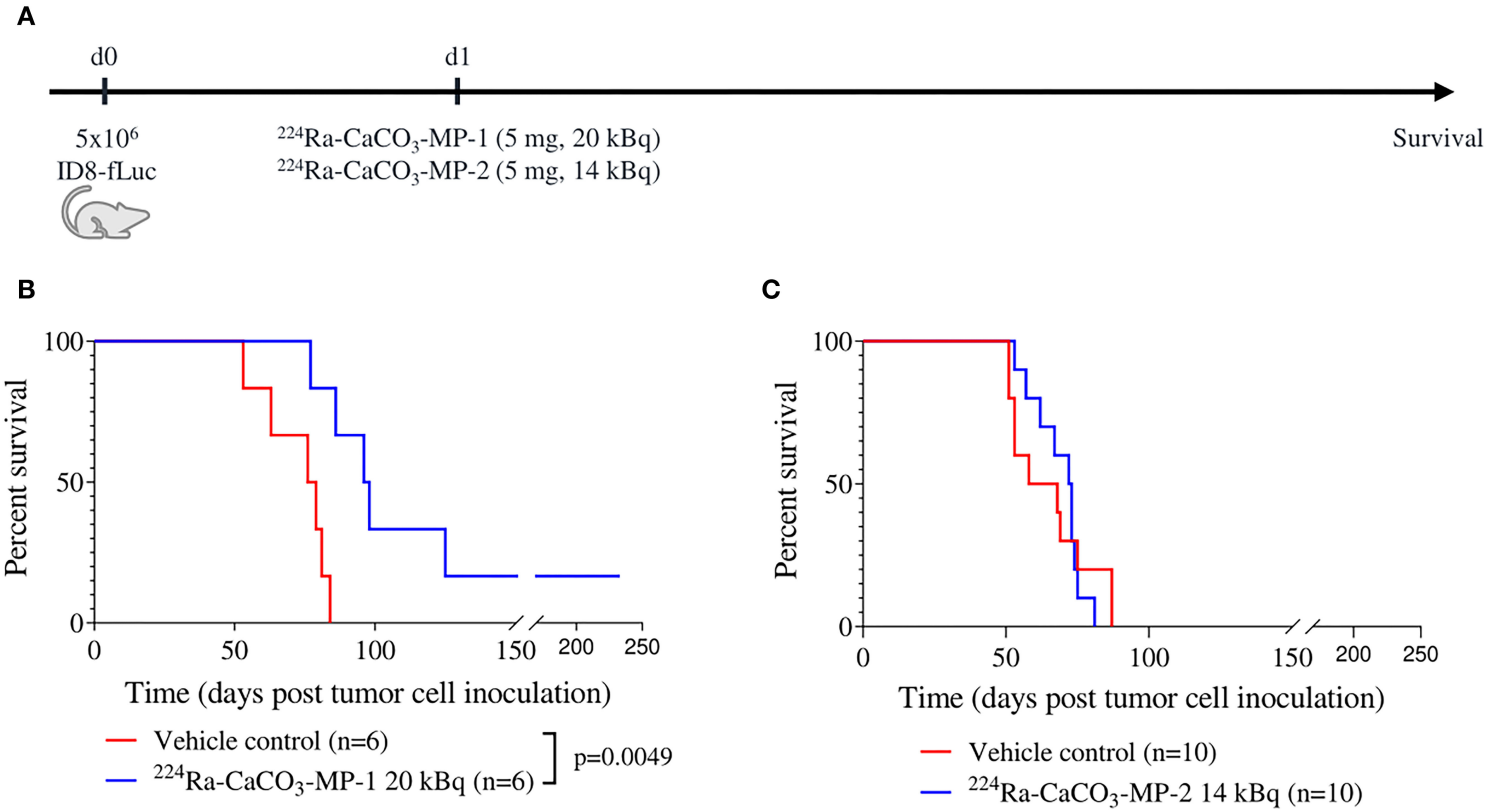
Experimental set-up **(A)** with corresponding Kaplan-Meier curves **(B,C)** for survival of mice injected IP with vehicle control and ^224^Ra-CaCO_3_-MP-1 (5 mg, 20 kBq) or ^224^Ra-CaCO_3_-MP-2 (5 mg, 14 kBq) on day 1 post tumor cell inoculation.

**Figure 2:**
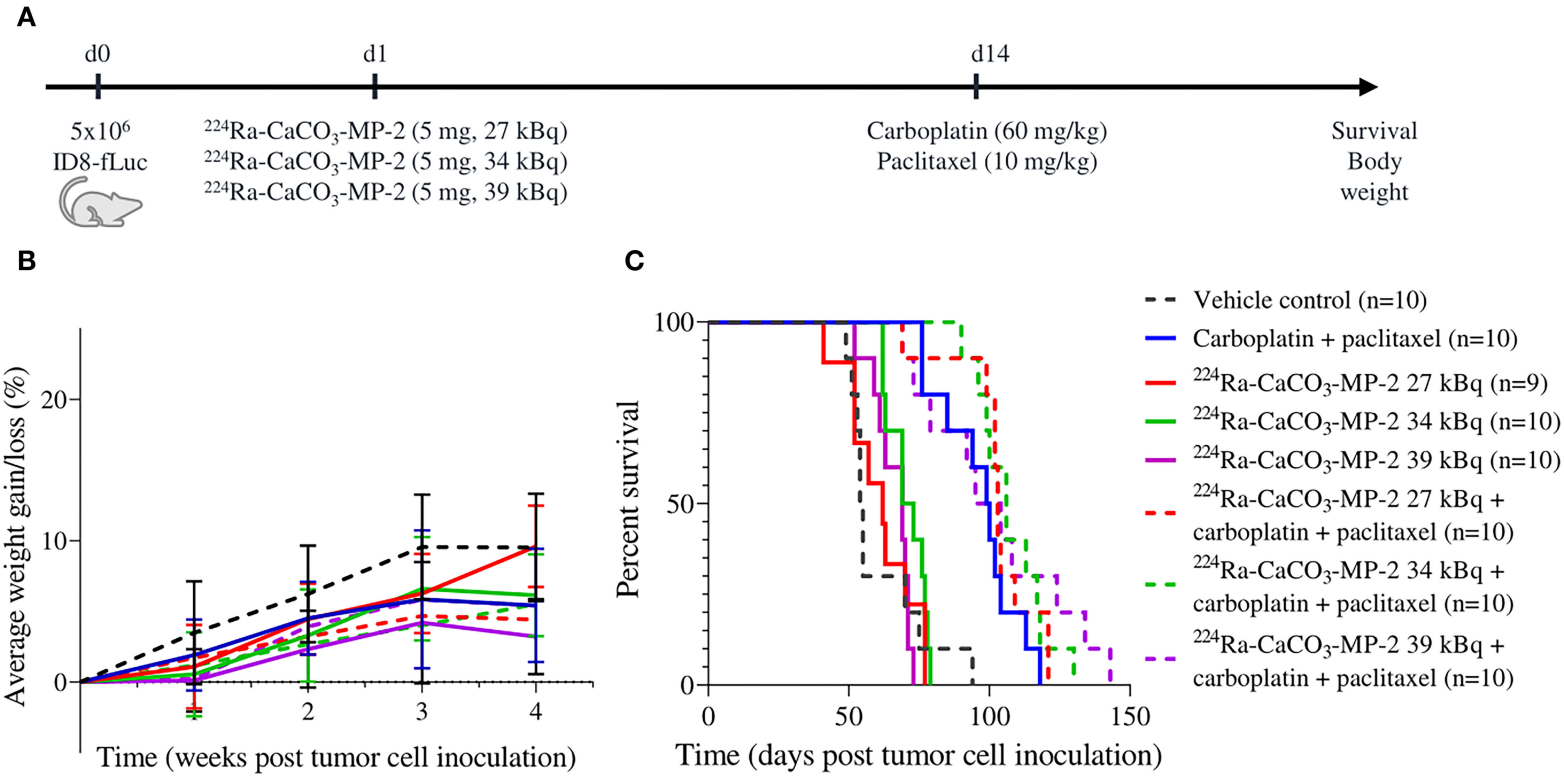
Experimental set-up **(A)** with corresponding body weight evolution as a percentage of starting weight **(B)** and Kaplan-Meier curve for survival **(C)** of mice injected IP with vehicle control, ^224^Ra-CaCO_3_-MP-2 (5 mg, 27/34/39 kBq) on day 1 post tumor cell inoculation and/or carboplatin (60 mg/kg) and paclitaxel (10 mg/kg) on day 14 post tumor cell inoculation. Due to procedural complications during tumor cell inoculation (injection in visceral peritoneum instead of peritoneal cavity), one animal allocated to the ^224^Ra-CaCO_3_-MP-2 27 kBq single treatment group was excluded from all further data analysis.

**Figure 3:**
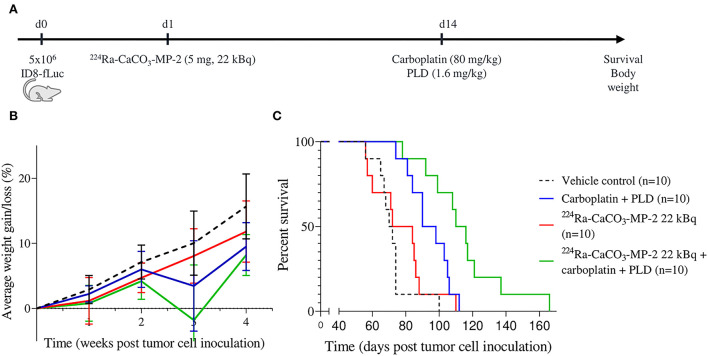
Experimental set-up **(A)** with corresponding body weight evolution as a percentage of starting weight **(B)** and Kaplan-Meier curve for survival **(C)** of mice injected IP with vehicle control, ^224^Ra-CaCO_3_-MP-2 (5 mg, 22 kBq) on day 1 post tumor cell inoculation and/or carboplatin (80 mg/kg) and PLD (1.6 mg/kg) on day 14 post tumor cell inoculation.

All ^224^Ra-CaCO_3_-MP treatments were administered on day 1 post tumor cell inoculation (Figures 1A, 2A, 3A). This time point was chosen to mimic minimal residual disease after a cytoreductive debulking surgery in patients, a situation highly relevant to target micro-metastatic disseminations in the peritoneal cavity. Chemotherapy administration in all experiments was performed at day 14 post tumor cell inoculation (Figures 2A, 3A), to mimic the adjuvant chemotherapy initiation in patients. All treatments were administered *via* IP injections. For all injection time points, control mice received either DPBS, 0.9% NaCl or Plasmalyte without additives as the appropriate vehicle control solution for their respective experimental treatment.

### Data analysis

A statistical power analysis was performed to determine sample sizes for all experiments. A power of at least 0.80 was reached with 6 to 10 mice per treatment group, depending on the type of experiment. Survival curves were compared using the log-rank (Mantel-Cox) test. Adjustment for multiple comparisons was performed with the Benjamini-Hochberg procedure (with Q = 5%). The comparisons made for the different experiments can be found in Supplementary Tables S1, S2. Median survival ratios (MSR) were calculated as the median survival of the experimental group divided by the median survival of the respective control group and served as an additional measure for efficacy. A linear mixed model was fitted to assess the effects of the ^224^Ra-CaCO_3_-MP on the weight changes of the mice in the first 4 weeks after treatment administration, with data points taken at 1 week intervals. An (adjusted) *p* < 0.05 was considered significant. Synergy between chemotherapy treatment and ^224^Ra-CaCO_3_-MP was assessed using the Bliss analysis method (29). For this, a Cox proportional-hazards model was fitted to the survival data. Synergy is evaluated based on the hazard ratios (HRs) of the interaction of groups treated with both chemotherapy and ^224^Ra-CaCO_3_-MP and the monotherapy treatment groups. Interaction values lower than 1 are considered synergistic, with statistical significance defined by a *p* < 0.05 and by the confidence interval not including 1.

The HR in case of synergy (HR_combination_) is calculated by multiplying the HRs of both single treatment and the HR of the interaction, and the HR in case of an additive effect (HR_additive_) is calculated by multiplying only the HRs of the single treatments.

The power analysis, Bliss analysis and linear mixed model fitting were performed using R version 4.1.0 (R Foundation for Statistical Computing, Vienna, Austria, https://www.R-project.org/), all other statistical analyses were performed using GraphPad Prism version 8.2.1 (GraphPad Software, San Diego, CA, USA).

## Results

### Therapeutic potential of ^224^Ra-CaCO_3_-MP as an IP treatment in the immune competent ID8-fLuc mouse model for ovarian cancer

Two product formulations of ^224^Ra-CaCO_3_-MP were evaluated in the immune competent ID8-fLuc mouse model for ovarian cancer. Treatment with the early-phase development product formulation ^224^Ra-CaCO_3_-MP-1 was able to significantly prolong survival compared to vehicle control mice (median survival of 97 and 77.5 days, respectively, *p* = 0.0049) and cured 17% of the mice at an activity dose of 20 kBq/mouse with an average of 1,004 kBq/kg body weight (Figure 1B). In a follow-up experiment with the ^224^Ra-CaCO_3_-MP-2 formulation developed for clinical use, where control of microparticle size over time was achieved and a sterilization procedure was introduced [20], there was no effect on overall survival at an activity dose of 14 kBq/mouse with an average of 805 kBq/kg body weight (Figure 1C). Even though the activity dose in this experiment was lower (14 compared to 20 kBq/mouse), higher activity doses that were used in the combination studies discussed below (ranging between 22 and 39 kBq/mouse) had a similar outcome (Figures 2C, 3C). The MSRs of all single treatments with ^224^Ra-CaCO_3_-MPs, were higher than 1 in all investigated conditions (Table 1).

**Table 1:** Median survival ratios (MSRs) for ^224^Ra-CaCO_3_-MP as a single treatment compared to vehicle control.

**Treatment (dose)**	**Median survival experimental group (days)**	**Median survival vehicle control group (days)**	**MSR**
*^224^Ra-CaCO_3_-MP-1 (5 mg, 20 kBq)*	97.0	77.5	1.3
*^224^Ra-CaCO_3_-MP-2 (5 mg, 14 kBq)*	72.5	63.0	1.2
*^224^Ra-CaCO_3_-MP-2 (5 mg, 22 kBq)*	78.0	71.0	1.1
*^224^Ra-CaCO_3_-MP-2 (5 mg, 27 kBq)*	62.5	54.5	1.1
*^224^Ra-CaCO_3_-MP-2 (5 mg, 34 kBq)*	71.0	54.5	1.3
*^224^Ra-CaCO_3_-MP-2 (5 mg, 39 kBq)*	69.0	54.5	1.3

### Therapeutic synergistic effect of ^224^Ra-CaCO_3_-MP combined with chemotherapy

^224^Ra-CaCO_3_-MPs were combined with two different chemotherapy regimens commonly used in clinical practice. In these studies, our first readout included therapeutic efficacy in terms of survival. In a first experiment we combined the first-line chemotherapy regimen carboplatin-paclitaxel with different activity doses of ^224^Ra-CaCO_3_-MP: 27, 34 and 39 kBq/mouse with an average of 1,466, 1,847 and 2,118 kBq/kg body weight. None of the activity dose levels of ^224^Ra-CaCO_3_-MP-2 in combination with carboplatin-paclitaxel were able to significantly improve survival compared to mice treated with carboplatin-paclitaxel alone (Figure 2C). No statistically significant synergistic effects were observed for any of the activity doses and the carboplatin-paclitaxel chemotherapy regimen. However, there is a tendency toward synergism for the highest activity dose level of ^224^Ra-CaCO_3_-MP-2 (39 kBq) when comparing the HR_combination_ (0.0667) with the HR_additive_ (0.1206). An overview of all HRs can be found in Table 2.

**Table 2:** Assessment of synergistic effect between ^224^Ra-CaCO_3_-MP-2 and carboplatin-paclitaxel or carboplatin-PLD.

^**224**^ **Ra-CaCO**_**3**_ **-MP-2 (5 mg, 27 kBq) with carboplatin (60 mg/kg)-paclitaxel (10 mg/kg)**			
**Treatment**	**Hazard ratio**	**95% CI**	***p-value***
Carboplatin-paclitaxel	0.0823	(0.0251–0.2691)	< 0.001
^224^Ra-CaCO_3_-MP-2	0.9125	(0.3617–2.3020)	0.846
Carboplatin-paclitaxel and ^224^Ra- CaCO_3_-MP-2	0.6020	(0.1609–2.2523)	0.451
HR_combination_	0.0452	na	na
HR_additive_	0.0751	na	na
^**224**^ **Ra-CaCO**_**3**_ **-MP-2 (5 mg, 34 kBq) with carboplatin (60 mg/kg)-paclitaxel (10 mg/kg)**			
Carboplatin-paclitaxel	0.0645	(0.0200–0.2080)	< 0.001
^224^Ra-CaCO_3_-MP-2	0.6153	(0.2444–1.5493)	0.303
Carboplatin-paclitaxel and ^224^Ra- CaCO_3_-MP-2	0.8600	(0.2339–3.1623)	0.820
HR_combination_	0.0341	na	na
HR_additive_	0.0397	na	na
^**224**^ **Ra-CaCO**_**3**_ **-MP-2 (5 mg, 39 kBq) with carboplatin (60 mg/kg)-paclitaxel (10 mg/kg)**			
Carboplatin-paclitaxel	0.1117	(0.0399–0.3132)	< 0.001
^224^Ra-CaCO_3_-MP-2	1.0801	(0.4141–2.8174)	0.875
Carboplatin-paclitaxel and ^224^Ra- CaCO_3_-MP-2	0.5525	(0.1404–2.1745)	0.396
HR_combination_	0.0667	na	na
HR_additive_	0.1206	na	na
^**224**^ **Ra-CaCO**_**3**_ **-MP-2 (5 mg, 22 kBq) with carboplatin (80 mg/kg) -PLD (1.6 mg/kg)**			
Carboplatin-PLD	0.2421	(0.0944–0.6208)	0.0032
^224^Ra-CaCO_3_-MP-2	0.5262	(0.2086–1.3273)	0.1738
Carboplatin-PLD and ^224^Ra- CaCO_3_-MP-2	0.5023	(0.1221–2.0658)	0.3399
HR_combination_	0.0640	na	na
HR_additive_	0.1274	na	na

PLD, pegylated liposomal doxorubicin; CI, confidence interval; na, not applicable.

Subsequently, we assessed a combination with carboplatin-PLD as an example of a second-line chemotherapy regimen. Only one activity dose of ^224^Ra-CaCO_3_-MP was included: 22 kBq/mouse with an average of 1,300 kBq/kg body weight. While ^224^Ra-CaCO_3_-MP-2 as a single treatment was not able to prolong survival, the combination of ^224^Ra-CaCO_3_-MP-2 combined with the carboplatin-PLD resulted in a prolonged survival compared to mice that received chemotherapy alone (median survival of 114 and 94.5 days, respectively, p_adj_ = 0.0102) (Figure 3C). However, the biologically observed synergistic effect between carboplatin-PLD and ^224^Ra-CaCO_3_-MP-2 treatment is not supported by a statistically significant effect (*p* = 0.3399), although there is a tendency toward synergism when comparing the HR_combination_ (0.0640) and the HR_additive_ (0.1274). An overview of the different HRs can be found in Table 2.

The MSRs were also determined for the combinations of ^224^Ra-CaCO_3_-MP-2 with the two different chemotherapy regimens, when compared to mice treated with chemotherapy alone. All MSRs ranged between 1 and 1.2 (Table 3).

**Table 3:** Median survival ratios (MSRs) for ^224^Ra-CaCO_3_-MP combined with chemotherapy compared to chemotherapy as a single treatment.

**Treatment (dose)**	**Median survival combination group (days)**	**Median survival chemotherapy only group (days)**	**MSR**
Carboplatin (60 mg/kg)-paclitaxel (10 mg/kg) and ^224^Ra-CaCO_3_-MP-2 (5 mg, 27 kBq)	103	99.5	1.0
Carboplatin (60 mg/kg)-paclitaxel (10 mg/kg) and ^224^Ra-CaCO_3_-MP-2 (5 mg, 34 kBq)	106	99.5	1.1
Carboplatin (60 mg/kg)-paclitaxel (10 mg/kg) and ^224^Ra-CaCO_3_-MP-2 (5 mg, 39 kBq)	99.5	99.5	1.0
Carboplatin (80 mg/kg)-PLD (1.6 mg/kg) and ^224^Ra-CaCO_3_-MP-2 (5 mg, 22 kBq)	113	94	1.2

PLD, pegylated liposomal doxorubicin.

### Combination of ^224^Ra-CaCO_3_-MPs with standard of care chemotherapy regimens is feasible in terms of tolerability

Changes in body weight over time was assessed as a measure of tolerability. From the body weight curves (Supplementary Figures S1, S2), there were no indications of persistent treatment related effects. A transient loss in body weight was observed in the days following both treatment with ^224^Ra-CaCO_3_-MP-2 and chemotherapy, with longest time to recovery (approximately 1 week) for the carboplatin and PLD regime. When body weight development over time was analyzed by fitting a linear mixed model, a statistically significant delay in body weight gain was found for the mice treated with the highest activity doses of 34 and 39 kBq/mouse compared to vehicle control mice (*p* = 0.047 and *p* < 0.001, respectively). However, ^224^Ra-CaCO_3_-MP-2 treatment was not inferior to chemotherapy treatment in terms of body weight development over time. If anything, treatment with carboplatin-paclitaxel resulted in a delayed weight progression compared to mice that received ^224^Ra-CaCO_3_-MP-2 at a dose of 27 kBq/mouse (p=0.045). More importantly, none of the groups receiving a combination of chemotherapy with the ^224^Ra-CaCO_3_-MP-2 treatment presented with delayed weight progression compared to mice that received chemotherapy alone, irrespective of the chemotherapy regimen (Figures 2B, 3B, Table 4). In addition, no apparent clinical signs of toxicity were observed in any of the mice in the duration of the studies.

**Table 4:** Overview of weight change assessment in mice that received ^224^Ra-CaCO_3_-MPs as a single treatment and in combination with both carboplatin-paclitaxel and carboplatin-PLD chemotherapy regimens.

^**224**^ **Ra-CaCO**_**3**_ **-MP-2 (5 mg, 22 kBq) vs. vehicle control**					
		**Estimate**	**SE**	**95% CI**	**p-value**
**Intercept**		−0.7547	0.3625	[−1.465; −0.044]	0.041
**Time**		2.9845	0.3951	[2.210; 3.759]	< 0.001
Time*treatment		0.9329	0.5319	[−0.110; 1.975]	0.097
^**224**^ **Ra-CaCO**_**3**_ **-MP-2 (5 mg, 27/34/39 kBq) vs. vehicle control**					
**Intercept**		−0.0792	0.2810	[−0.632; 0.473]	0.779
**Time**		2.5870	0.2699	[2.086; 3.102]	< 0.001
Time*treatment	27 kBq	−0.1453	0.3904	[−0.890; 0.577]	0.712
	34 kBq	−0.7783	0.3800	[−1.501; −0.073]	0.047
	39 kBq	−1.5413	0.3800	[−2.262; −0.835]	< 0.001
^**224**^ **Ra-CaCO**_**3**_ **-MP-2 (5 mg, 22 kBq) with carboplatin (80 mg/kg)-PLD (1.6 mg/kg) vs. carboplatin-PLD**					
**Intercept**		−0.1514	0.7267	[−1.576; 1.273]	0.836
**Time**		1.2681	0.4324	[0.421; 2.116]	0.006
Time*treatment		0.8688	0.5065	[−0.124; 1.862]	0.103
^**224**^ **Ra-CaCO**_**3**_ **-MP-2 (5 mg, 27/34/39 kBq) with carboplatin (60 mg/kg)-paclitaxel (10 mg/kg) vs. carboplatin-paclitaxel**					
**Intercept**		0.1806	0.2556	[−0.322; 0.683]	0.481
**Time**		1.5512	0.3093	[0.974; 2.133]	< 0.001
Time*treatment	27 kBq	−0.3239	0.4323	[−1.133; 0.484]	0.458
	34 kBq	−0.2156	0.4323	[−1.027; 0.590]	0.621
	39 kBq	0.0202	0.4323	[−0.792; 0.825]	0.963
^**224**^ **Ra-CaCO**_**3**_ **-MP-2 (5 mg, 22 kBq) vs. carboplatin (80 mg/kg)-PLD (1.6 mg/kg)**					
**Intercept**		−0.3848	0.4784	[−1.322; 0.553]	0.424
**Time**		2.2147	0.3974	[1.436; 2.994]	< 0.001
Time*treatment		0.6465	0.5148	[−0.363; 1.656]	0.225
^**224**^ **Ra-CaCO**_**3**_ **-MP-2 (5 mg, 27/34/39 kBq) vs. carboplatin (60 mg/kg)-paclitaxel (10 mg/kg)**					
**Intercept**		−0.1183	0.2762	[−0.665; 0.428]	0.670
**Time**		1.5848	0.2781	[1.049; 2.129]	< 0.001
Time*treatment	27 kBq	0.8301	0.3994	[0.046; 1.606]	0.045
	34 kBq	0.2130	0.3887	[−0.543; 0.963]	0.587
	39 kBq	−0.5364	0.3887	[−1.287; 0.210]	0.176

Statistical analysis was performed by fitting a linear mixed model.

PLD, pegylated liposomal doxorubicin; SE, standard error; CI, confidence interval.

## Discussion

In the search for more effective treatment strategies for ovarian cancer, α-emitting radionuclide therapies are emerging. The high energy deposition in combination with limited penetration depth can be exploited to target residual microscopic disease without affecting the surrounding radiosensitive organs. These micrometastases remain present within the peritoneal cavity after cytoreductive debulking surgery and are often related to the high recurrence rate of the disease. In this study, we specifically investigated the therapeutic potential of a newly developed α-emitting radiopharmaceutical which consists of ^224^Ra adsorbed onto CaCO_3_ microparticles, and the safety to use this in combination with chemotherapy regimens commonly used in clinical practice. We provide proof-of-principle of the therapeutic potential of ^224^Ra-CaCO_3_-MPs in a syngeneic model of ovarian cancer in immune competent mice. Furthermore, the sequential administration of ^224^Ra-CaCO_3_-MPs with two different standard of care chemotherapy regimens indicated that a synergistic effect can be obtained, however, the synergism was more pronounced with carboplatin-PLD compared to carboplatin-paclitaxel. In general, the various treatment combinations appeared well-tolerated in the mice.

The therapeutic potential of ^224^Ra-CaCO_3_-MPs in the immune compromised ES-2 and SKOV3 mouse model for ovarian cancer have previously been demonstrated. Different product formulations of ^224^Ra-CaCO_3_-MPs were able to prolong survival with MSRs ranging between 1.5 and 2.8 (20, 21, 28), while the MSRs observed in the current study ranged between 1.1 and 1.3. An important factor that might negatively influence the therapeutic efficacy in the immune competent ID8-fLuc mouse model for ovarian cancer is the reaction of the tumor immune microenvironment to the particle drug carrier (CaCO_3_ microparticles). It has been shown that IP injections of microparticle drug carriers, including but not limited to CaCO_3_ microparticles, elicit an immune suppressive and tumor promoting effect in the ID8-fLuc model, mediated by innate immune suppressive cells such as myeloid-derived suppressor cells and M2-like macrophages (30). Both cell types are known to be involved in ovarian cancer development and progression [recently reviewed (31)]. We believe that the slight survival benefit in the ID8-fLuc model can be explained by the fact that the immune-related tumor promoting mechanisms in response to the CaCO_3_ microparticles partially counteract the therapeutic effect of the ^224^Ra. In another syngeneic mouse model of disseminated peritoneal disease, albeit of colorectal origin (CT26.WT), treatment with the ^224^Ra-CaCO_3_-MPs was able to significantly prolong survival (MSR of 1.8) (28), indicating that the tumor-promoting mechanisms are not universal among different disease models.

One novelty with the current study is the use of the fully immune competent ID8-fLuc mouse model for ovarian cancer. Previously published work on the ^224^Ra-CaCO_3_-MPs in ovarian cancer models was performed in immune compromised mouse models (ES-2 and SKOV3). Since the strong immune suppressive tumor microenvironment in patients with ovarian cancer is an important factor in the disease progression (31), we provide additional proof of the therapeutic potential of the ^224^Ra-CaCO_3_-MPs in a mouse model that closely resembles this clinical situation. The study design was aimed to mimic the clinically relevant standard of care chemotherapy regimens, although, it should be noted that the IP administration route of the different chemotherapeutics differs from the standard administration route in patients with ovarian cancer (intravenous administration).

However, we recognize that our study encounters some limitations. With the current data, we are not able to provide a mechanistic explanation as to why two chemotherapy chemotherapy regimens result in a different outcome when combined with ^224^Ra-CaCO_3_-MPs. Several mechanisms can be responsible for creating synergistic or additive effects between chemotherapy and radiation therapy. The mechanism of action for the specific chemotherapeutic drug may radiosensitize tumor cells to α-radiation to a varying degree. In addition, it is known that different chemotherapy regimens have different effects on the ovarian cancer immune microenvironment in mice (32, 33). Hence, the immune response caused by the ^224^Ra-CaCO_3_-MPs and the chemotherapeutics may favor some but not all combinations and dosages. A future characterization of both the cytotoxic mechanisms and immunological responses of the combined ^224^Ra-CaCO_3_-MPs and chemotherapy treatment might therefore aid with identifying the most optimal combination regimens.

In the past, other applications with α-particle emitters have been evaluated for the treatment for ovarian cancer. Preclinical evaluation of IP treatment with ^211^At-labeled monoclonal antibodies showed a high therapeutic efficacy in treating micrometastatic growth in the OVCAR-3 ovarian cancer mouse model (34, 35). Additionally, the α-emitter ^212^Pb has been evaluated as an IP treatment in the immunodeficient ES-2 and A2780cp20 mouse models for ovarian cancer showing a therapeutic potential when labeled to a monoclonal antibody or CaCO_3_ microparticles (36, 37). Additionally, ^212^Pb and ^211^At colloids have also been investigated previously in a preclinical setting for IP ovarian cancer dissemination, where they have proven their therapeutic potential (38, 39). No immediate and/or late signs of local radiation-induced toxicities were observed in the phase I clinical evaluation of ^211^At- or ^212^Pb-labeled antibody treatments in patients with ovarian cancer (18, 19, 40, 41). These results are as expected with the limited range of tissue penetration of α-emitters, preventing irradiation of other radiosensitive organs within the peritoneal cavity.

Furthermore, the combined effects of α-therapies and chemotherapeutics on weight development in mice as a measure for toxicity have been evaluated previously. Milenic and colleagues reported a modest weight loss in mice treated sequentially with ^212^Pb-trastuzumab and gemcitabine compared to mice that received gemcitabine alone in a model for colon carcinoma (LS-174T) (42), which is in contrast to what we observed in our study. However, the same group reported no difference in weight development between mice that received paclitaxel and ^213^Bi-trastuzumab or paclitaxel alone in the same tumor model (43), indicating a differential response to combinations of different types and dosages of chemotherapy and radionuclides. Both combination regimens described above also produced synergistic therapeutic effects that could not be reached by these therapeutics separately (42, 43).

We provide proof-of-principle for the therapeutic efficacy ^224^Ra-CaCO_3_-MPs in an immune competent mouse model for ovarian cancer, both alone and in combination with chemotherapy. Furthermore, the results indicate a safe sequential administration with two different chemotherapy regimens often used in clinical practice. The results support further evaluation of ^224^Ra-CaCO_3_-MPs in patients with ovarian cancer. However, further investigations remain to identify the most optimal chemotherapy regimen to combine with ^224^Ra-CaCO_3_-MPs and the sequence of therapies to fully exploit a potential synergistic effect.

## Data availability statement

The raw data supporting the conclusions of this article will be made available by the authors, without undue reservation.

## Ethics statement

The animal study was reviewed and approved by Katholieke Universiteit Leuven.

## Author contributions

Conceptualization and interpretation of results: RW, SW, AC, and TB. Design of experiments: RW and SW. *In vivo* experiments: RW, YB, and MR. Analysis of results: RW and MR. Statistics: RW and JC. Manuscript writing: RW. Manuscript proof-reading: JC, YB, MR, SW, TB, IV, and AC. Supervision: SW, TB, IV, and AC. Funding acquisition: TB and AC. All authors have read and agreed to the published version of the manuscript.

## Funding

This research was funded by the Norwegian Research Council (with project number 304591) and Oncoinvent AS.

## Conflict of interest

RW was employed by Oncoinvent AS. AC was a contracted researcher for Oncoinvent AS and Novocure and a consultant for Sotio a.s. SW was employed by and a shareholder of Oncoinvent AS. TB was employed by and a shareholder of Oncoinvent AS. IV is a consultant for Agenus, Akesobio, AstraZeneca, Bristol Myers Squibb, Deciphera Pharmaceuticals, Eisai, Elevar Therapeutics, F. Hoffmann-La Roche, Genmab, GSK, Immunogen, Jazzpharma, Karyopharm, Mersana, MSD, Novocure, Novartis, Oncoinvent, OncXerna, Sanofi, Seagen, Sotio, Verastem Oncology and Zentalis, was a contracted researcher for Oncoinvent AS, performs corporate sponsored research for Amgen and Roche, and receives accommodation and travel expenses from Karyopharm, Genmab and Novocure. The funders (The Norwegian Research Council (project 304591) and Oncoinvent AS) provided support in the form of salaries for authors RW, SW, and TB but did not have any additional role in the study design, data collection, analysis or interpretation, preparation of the manuscript, or decision to publish. The remaining authors declare that the research was conducted in the absence of any commercial or financial relationships that could be construed as a potential conflict of interest.

## Publisher's note

All claims expressed in this article are solely those of the authors and do not necessarily represent those of their affiliated organizations, or those of the publisher, the editors and the reviewers. Any product that may be evaluated in this article, or claim that may be made by its manufacturer, is not guaranteed or endorsed by the publisher.
