# Supplementary material for: Intraperitoneal alpha therapy with 224Ra-labeled microparticles combined with chemotherapy in an ovarian cancer mouse model
Source: Front Med (Lausanne). 2022 Oct 10;9:995325. doi: 10.3389/fmed.2022.995325 (PMC9588927; doi:10.3389/fmed.2022.995325)
Supplement: Supplementary file 1 [file Data_Sheet_1.PDF]

Table S1. Overview of multiple comparisons made with the Benjamini-Hochberg procedure (with Q=5%) with corresponding adjusted p-values for the experiment where ^{224}Ra-$CaCO_3$-MP-2 treatment was combined with carboplatin-PLD chemotherapy.

	Vehicle control	Carboplatin-PLD	^{224}Ra-$CaCO_3$-MP-2	^{224}Ra-$CaCO_3$-MP-2 + carboplatin-PLD
Vehicle control	/	/	0,3770	0,0006
Carboplatin-PLD	/	/	0,0679	0,0102
^{224}Ra-$CaCO_3$-MP-2	0,3770	0,0679	/	0,0015
^{224}Ra-$CaCO_3$-MP-2 + carboplatin-PLD	0,0006	0,0102	0,0015	/

Table S2. Overview of multiple comparisons made with the Benjamini-Hochberg procedure (with Q=5%) with corresponding adjusted p-values for the experiment where ^{224}Ra-$CaCO_3$-MP-2 treatment was combined with carboplatin-paclitaxel chemotherapy.

	Vehicle control	Carboplatin-paclitaxel	^{224}Ra-$CaCO_3$-MP-2 27 kBq	^{224}Ra-$CaCO_3$-MP-2 34 kBq	^{224}Ra-$CaCO_3$-MP-2 39 kBq	^{224}Ra-$CaCO_3$-MP-2 27 kBq + carboplatin-paclitaxel	^{224}Ra-$CaCO_3$-MP-2 34 kBq + carboplatin-paclitaxel	^{224}Ra-$CaCO_3$-MP-2 39 kBq + carboplatin-paclitaxel
Vehicle control	/	0,0007	0,6174	0,5003	0,7181	/	/	/
Carboplatin-paclitaxel	0,0007	/	/	/	/	0,6203	0,5003	0,7181
^{224}Ra-$CaCO_3$-MP-2 27 kBq	0,6174	/	/	/	/	/	/	/
^{224}Ra-$CaCO_3$-MP-2 34 kBq	0,5003	/	/	/	/	/	/	/
^{224}Ra-$CaCO_3$-MP-2 39 kBq	0,7181	/	/	/	/	/	/	/
^{224}Ra-$CaCO_3$-MP-2 27 kBq + carboplatin-paclitaxel	/	0,6203	/	/	/	/	/	/
^{224}Ra-$CaCO_3$-MP-2 34 kBq + carboplatin-paclitaxel	/	0,5003	/	/	/	/	/	/
^{224}Ra-$CaCO_3$-MP-2 39 kBq + carboplatin-paclitaxel	/	0,7181	/	/	/	/	/	/

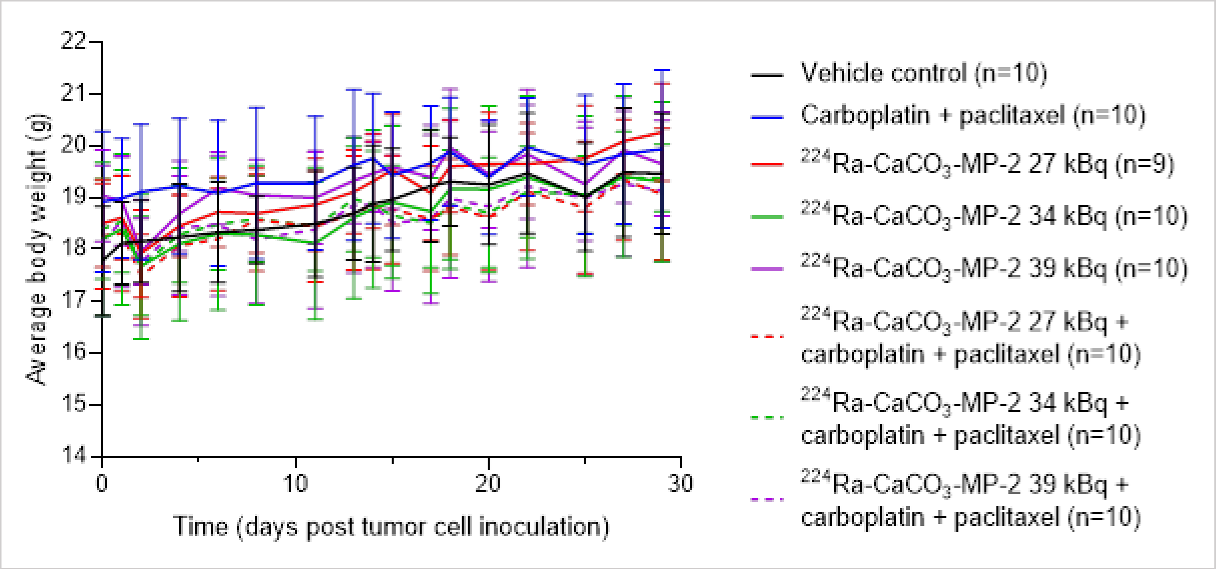

Figure S1. Average body weight development over time of mice injected IP with vehicle control, ^{224}Ra-$CaCO_3$-MP-2 (5 mg, 27/34/39 kBq) on day 1 post tumor cell inoculation and/or carboplatin (60 mg/kg) and paclitaxel (10 mg/kg) on day 14 post tumor cell inoculation. Due to procedural complications during tumor cell inoculation (injection in visceral peritoneum instead of peritoneal cavity), one animal allocated to the ^{224}Ra-$CaCO_3$-MP-2 27 kBq single treatment group was excluded from all further data analysis.

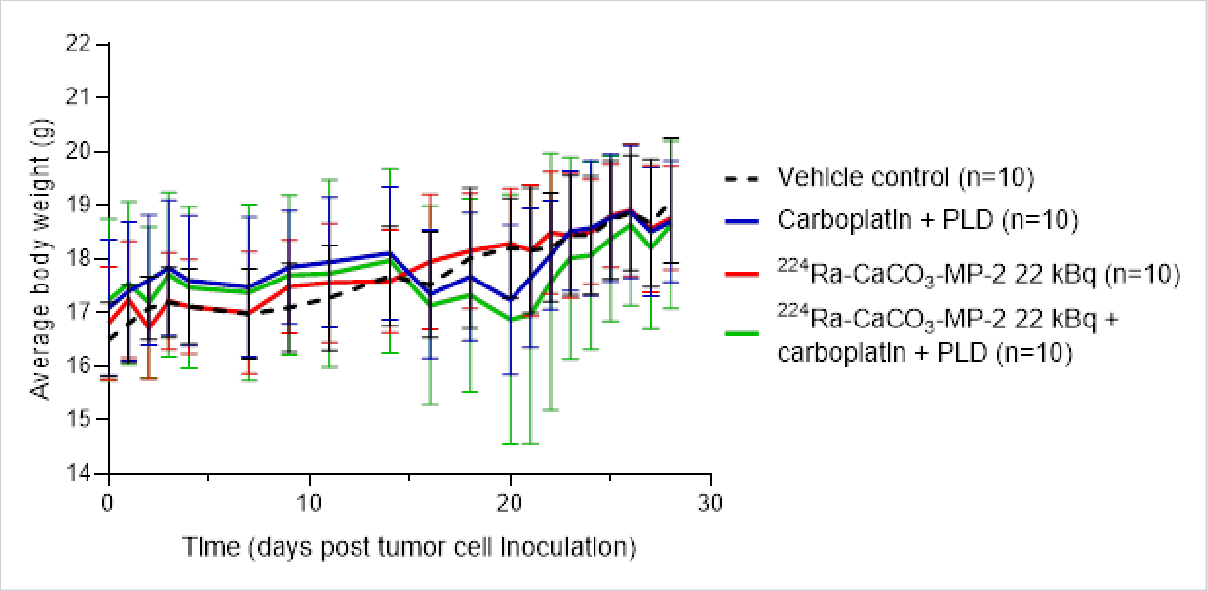

Figure S2. Average body weight development over time of mice injected IP with vehicle control, ^{224}Ra-$CaCO_3$-MP-2 (5 mg, 22 kBq) on day 1 post tumor cell inoculation and/or carboplatin (80 mg/kg) and PLD (1.6 mg/kg) on day 14 post tumor cell inoculation.
